# Changes in 3-Dimensional Measurements of Masseter Muscle After Orthognathic Surgery in Patients with Facial Asymmetry

**DOI:** 10.1007/s00266-024-04309-2

**Published:** 2024-08-26

**Authors:** Xiaobin Yang, Zhengguo Piao, Yaoran Liu, Lunqiu Chen, Luo Huang

**Affiliations:** 1 Department of Oral and Maxillofacial Surgery, Guangzhou Key Laboratory of Basic and Applied Research of Oral Regenerative Medicine, Affiliated Stomatology Hospital of Guangzhou Medical University, Guangzhou, 510150 China; 2 Key Laboratory of Oral Medicine, Guangzhou Institute of Oral Disease, NO.39 Huangsha Avenue, Guangzhou, 510150 China

**Keywords:** Facial asymmetry, Masseter muscle, Orthognathic surgery, Three-dimensional

## Abstract

**Objective:**

The aim of this study was to quantitatively evaluate morphological and volumetric changes in the masseter muscle using 3-dimensional analysis of facial asymmetry patients and to identify factors influencing these changes before and after orthognathic surgery.

**Methods:**

[Reviewer1 (2)]A single-center retrospective cohort study was conducted on twenty-two patients with deviation of the chin > 4 mm. Masseter muscle volume and morphology were measured at different periods during long-term follow-up (mean 15 ± 3.2 months). Factors related to changes in masseter muscle cross-sectional area and volume analyzed were also analyzed.

**Results:**

[Reviewer1 (2) and (9)]The volume and cross-sectional area of the masseter muscle on the non-deviated side reduced by 13.5% (*P* < 0.05) and 16.4% (*P* < 0.05), respectively, after orthognathic surgery. The length of the masseter muscle increased by 13.9% on the deviated side (*P* < .05) but decreased by 11.7% on the non-deviated side (*P* < 0.05). The width decreased on the deviated side from T1 to T2 (13.51 ± 2.09 mm vs. 12.04 ± 1.39 mm), but the non-deviated side showed an opposite tendency (10.81 ± 1.31 mm vs. 12.69 ± 2.37 mm). The difference in masseter muscle length and width between the two sides significantly reduced after surgery (*P* < 0.05). There was a noticeable decrease in the asymmetry in the muscle in proportion to the degree of the occlusal plane angle.

**Conclusion:**

Masseter muscle asymmetry exists in patients with facial asymmetry, but it could be improved with maxilla-mandible correction. Atrophy of the masseter muscle after orthognathic surgery was greater in patients with a large inclined occlusal plane angle due to improved dental compensation.

**Level of Evidence IV:**

This journal requires that authors assign a level of evidence to each article. For a full description of these Evidence-Based Medicine ratings, please refer to the Table of Contents or the online Instructions to Authors www.springer.com/00266.

## Introduction

Facial asymmetry can cause malocclusion and interfere with proper jaw movement, as well as affect individual’s appearance. Although asymmetry can occur in any region of the face and vary in severity, it is observed more often in the mandibular region and lower third of the face [1]. According to previous studies, changes in the underlying masseter muscle morphology could be a factor causing morphological changes in the buccal area of the face [2, 3]. Therefore, the relationship between the masseter muscles and maxillofacial morphology has been studied. Individuals with wider and shorter faces have strong mandibular muscles [4]. The morphology of the masseter muscle in patients with mandibular prognathism significantly differs from that in normal [5]. Patients with facial asymmetry experience asymmetry in the movement of muscles responsible for stomatognathic functions [6, 7]. In addition, the results of some studies have indicated that the masseter muscle strongly impacts craniofacial growth and development. Resection of the masseter muscle significantly influences mandibular morphology, including the condylar area, angle, and development direction of the jaw [8].

Orthognathic surgery is a common treatment used to correct facial asymmetry. It can successfully change the position of bony structures and greatly enhance symmetry of the face. Surgical procedures commonly performed for the correction of asymmetry require three-dimensional repositioning of the maxillomandibular complex, which is challenging for the surgeon. Skeletal stability must be maintained within the physiological limits of the temporomandibular joint (TMJ). Changes in skeletal stability and occlusion can induce changes in the masticatory force vectors and occlusal force. To our knowledge, the masseter muscles play an important role in masticatory function. This is one of the most important factors affecting mandibular movement, occlusion, and postoperative stability. It has been reported that the masseter muscle can be strongly related to occlusal force [9]. Patients with dentofacial deformities have less bite force value than normal occlusion patients do [5]. However, the occlusal force increased after orthognathic surgery and the maximal bite force remained unchanged [10]. Several studies have shown that patients with facial asymmetry experience significant alterations in the masseter muscles, jaw biting force, and occlusion after orthognathic surgery [11–14]. There is still no sufficient analysis of masseter muscle changes and occlusal changes. There are no studies that describe both the morphological and volumetric changes of the masseter muscle and their relationship with orthognathic surgery in patients with facial asymmetry. The relationship between morphology and function is very important to synthesize various information about how much muscle force reflects the occlusal force. The purpose of the current study was to investigate morphological and volumetric changes in the masseter muscles and the potential factors that influence these changes before and after orthognathic surgery in patients with facial asymmetry

## Materials and Methods

### Study Design and Patients

To analyze changes in the masseter muscles after orthognathic surgery, we designed and implemented a single-center retrospective longitudinal study. The study group consisted of 22 Chinese patients (4 men and 18 women; mean age, 22.9 years range, 19–36 years). The included patients had dentofacial deformities with mandibular asymmetry that showed a deviation of the chin (Menton) greater than 4 mm from the facial midline [15]. All patients underwent sagittal split ramus osteotomy with Le Fort I osteotomy at the Department of Oral and Maxillofacial Surgery of the Affiliated Stomatological Hospital of Guangzhou Medical University between January 2019 and December 2022 by one operator. Patients of non-Chinese nationality, with other genetic disorders, facial skeletal disorders, a history of prominent fractures, or who had undergone head and neck surgeries were excluded. This study was conducted in accordance with the Declaration of Helsinki and approved by the Institutional Review Board at the Affiliated Stomatological Hospital of Guangzhou Medical University. The institutional review board number is LCYJ2024002. Written informed consent was obtained from each patient enrolled in the study, and each patient was allowed to withdraw from the study at any time.

### Measurements with Three-Dimensional Computed Tomography

CBCT scans were taken with the mandible in the resting position before surgery and at 1 year after surgery with the Newtom VGi (Quantitative Radiology, Verona, Italy). The scans were conducted with the following settings: field of view of 24 × 19 cm, 90 kV, 6.0 mA, scan time of 15 s, and voxel size of 0.3 mm. The CBCT scans were exported in DICOM files and measurements were taken using Mimics 21.0 software (Materialise, Leuven, Belgium), which allows for the reconstruction of both the craniofacial skeleton and the patient’s soft tissue. To standardize the CBCT scans, the same operator oriented the head position in the virtual space according to the transverse, coronal, and sagittal reference planes [16].

**Fig. 1:**
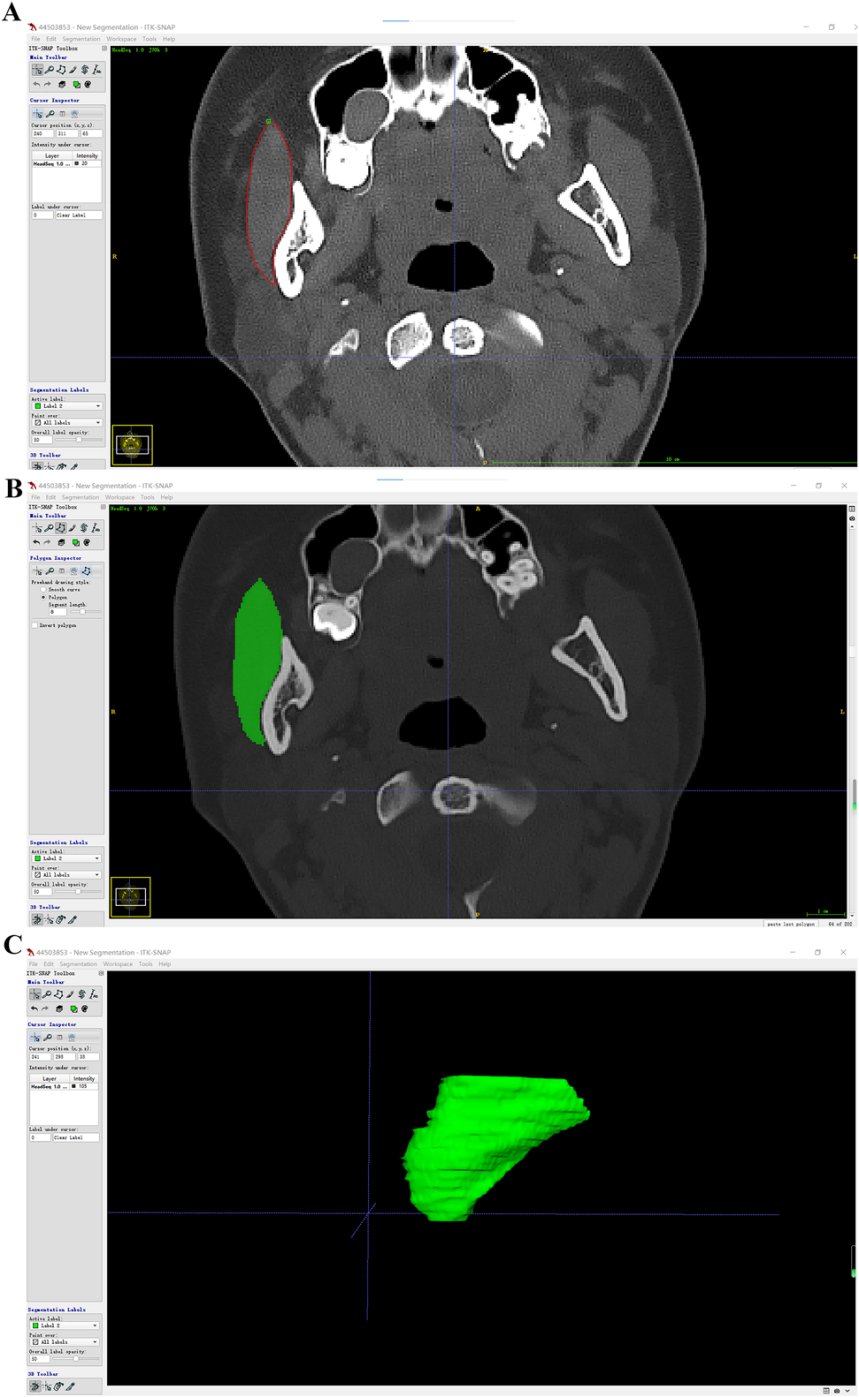
Sketch map of three-dimensional reconstruction of masseter muscle. Red represents the left masseter and green represents the right masseter

To avoid confusion between the masseter muscle and the medial and lateral pterygoid muscles extending beyond the sigmoid notch, we selected the region of the masseter muscle beneath the sigmoid notch of the mandible as our measurement area. To differentiate the masseter muscle from bone and other soft tissues, we performed a manual selection layer by layer using ITK-SNAP 3.6.0 (http://www.itksnap.org). Then, a 3D image of the masseter muscle was obtained after calculation for further measurement (Fig.1). The figure station exported the volume automatically.

**Fig. 2:**
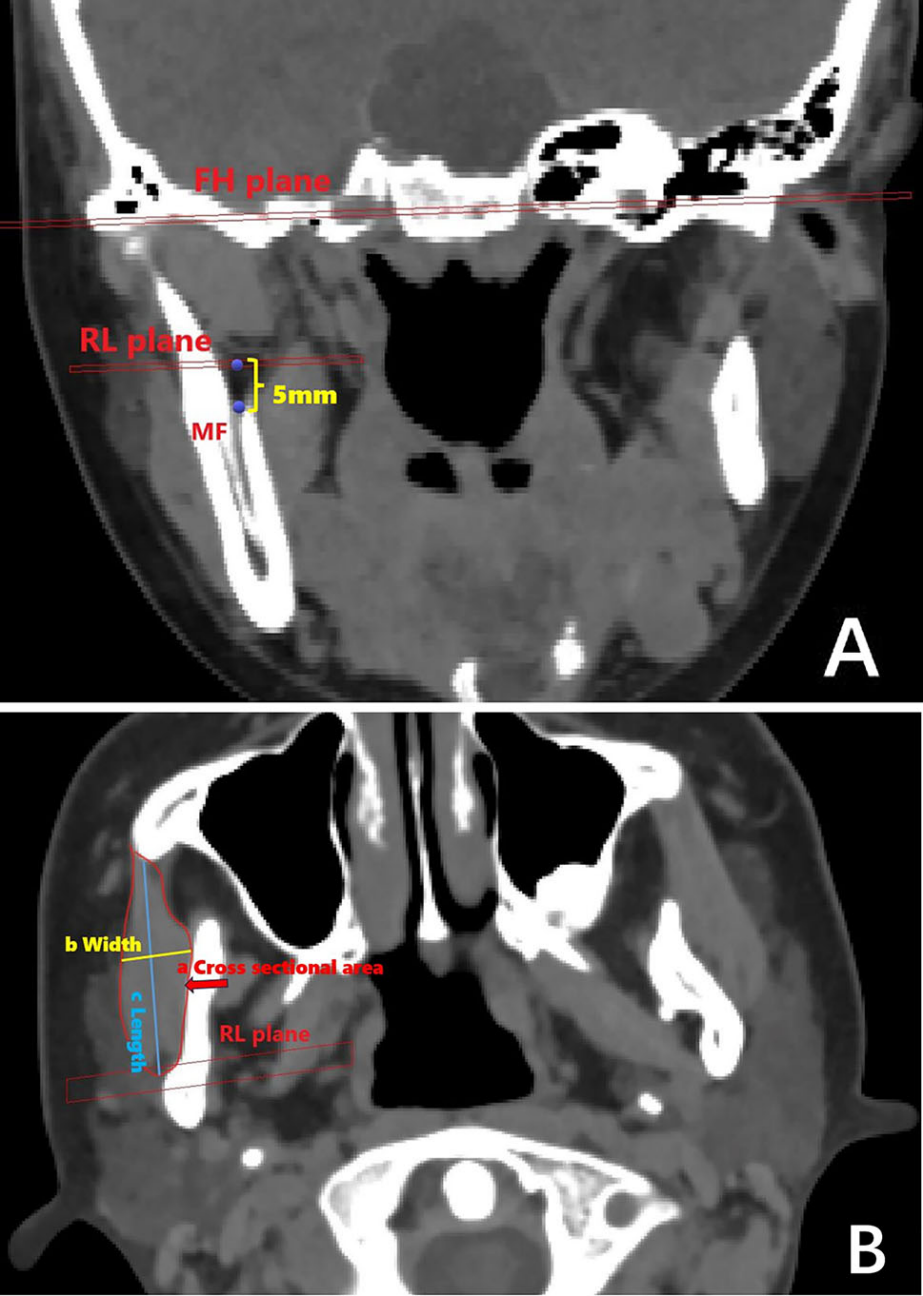
**A** RL plane: 5 mm above the mandibular foramen (MF) and parallel to the FH plane; **B** A tomograph image that demonstrates a) masseter muscle area: square of masseter muscle b) masseter muscle width: the thickest distance of the masseter muscle, c) masseter muscle length: the distance between the most anterior point and most posterior point of the masseter muscle

**Fig. 3:**
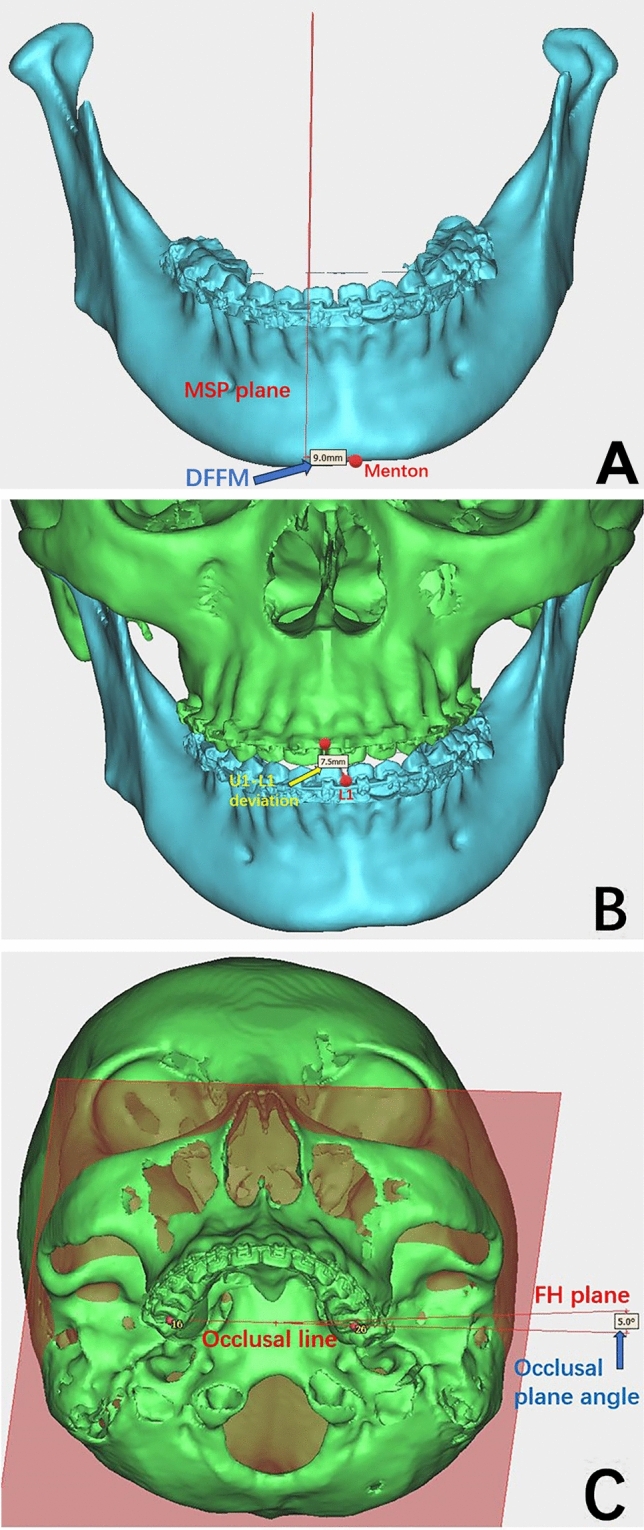
Factors related to morphological masseter muscle changes **A** DFFM: deviation from the facial midline; **B** Ul-Ll deviation: deviation between Ul midpoint of the upper incisor edge and LI midpoint of the lower incisor edge; **C** occlusal plane angle: angle between the occlusal line (a line connecting the midpoint of the left first maxillary molar and the right first maxillary molar) and FH plane

The image was reoriented using 2 reference planes: the Frankfort horizontal plane (FH), which passed through the bilateral orbitals and left portion, and the mid-sagittal plane (MSP), which passed through the nasion and sella and was perpendicular to the FH. The side of the lateral Menton deviation associated with the MSP was defined as the deviated side (Dev side), and the opposite side was defined as the non-deviated side (N-Dev side). Measurements were taken on the level of RL plane, which was 5 mm above the mandibular foramen (MF) and parallel to the FH plane [12] (Fig. 2A). The cross-sectional area and morphology of the masseter muscle were measured and assessed using previously described methods [13, 17] (Fig. 2B). Furthermore, factors related to morphological changes of the masseter muscles, including deviation from facial midline (DFFM) (Fig. 3A), Ul-Ll deviation (Fig. 3B), occlusal plane angle (Fig. 3C), were also investigated. Among these factors, 18 patients had occlusal plane angle greater than 3 degrees, accounting for approximately 81.8% of the total number of patients. All measurements were taken twice by 2 independent investigators, with a 7-day interval, and the average of the measured values was calculated and used in subsequent analyses.

## Statistical Analysis

Univariate analysis of variance for repeated measures was performed. Changes in the above-described measurements were assessed after orthognathic surgery. Preoperative and postoperative values, given as mean (SD), were compared using the paired t tests, and the changes were also calculated. Linear regression was used to analyze the associations between the cross-sectional area and the volume of the masseter muscle and other factors. All tests were 2-sided, and *P* values < 0.05 were considered significant.

## Results

All the patients tolerated the operation well, and no signs of infection or other complications were noted during or after the operation. None of the patients experienced any obvious difficulty with mastication.

### Changes after Orthognathic Surgery

One year postoperatively, the masseter muscle volume on the deviated side and the non-deviated side reduced by 11.8% and 16.4%, respectively, but only the volume on the non-deviated side was statistically significant (*P* < 0.05) (Table 1). The changes in the cross-sectional area were similar to those in the masseter muscle volume. The length and width of the masseter muscle on both sides significantly changed from T1 to T2 (*P *< 0.05) (Table 1). The masseter muscle was shorter on the deviated side (38.96 ± 3.83 mm) than on the non-deviated side (46.18±3.49 mm) before surgery, but was longer on the deviated side (46.18±3.49 mm) than on the non-deviated side (42.98 ± 3.26 mm) after surgery (Table 2). The width of deviated side decreased from T1 to T2 (13.51 ± 2.09 mm vs 12.04 ± 1.39 mm), but the non-deviated side showed an opposite tendency (10.81 ± 1.31 mm vs 12.69 ± 2.37 mm) (Table 2). However, there were no significant differences in the volume or cross-sectional area of the masseter muscle between the non-deviated side and deviated side before and after surgery. The differences between the two sides exhibited reduction postoperatively. The differences in the length and width of the masseter muscles on both sides significantly changed from T1 to T2 (*P* < 0.05) (Table 2).

**Table 1:** Changes in masseter muscle 1 year after orthognathic surgery

Variable	Dev		N-Dev	
	Mean Change in Value	*P-*value	Mean Change in Value	*P-*value
Masseter muscle volume (mm^3^ )	− 1665.37 (11.8%)	0.102	− 2581.58 (16.4%)	0.02*
Masseter muscle cross-sectional area (mm^2^)	− 28.06 (7.1%)	0.191	− 54.68 (13.5%)	0.01*
Masseter muscle length (mm)	5.43 (13.9%)	<0.001*	− 5.4 (11.7%)	< 0.001*
Masseter muscle width (mm)	− 1.47 (10.9%)	0.009*	1.88 (17.4%)	0.003*

^*^p < 0.05 paired *t* test

**Table 2:** Comparison of masseter muscle between deviated and non-deviated side before (T1) and 1 year after (T2) orthognathic surgery

Variable	T1			T2			T1-T2
	Dev	N-Dev	*P-*value	Dev	N-Dev	*P-*value	*P-*value
Masseter muscle volume (mm^3^)	14056.70 ± 3524.54	15772.30 ± 3701.60	0.123	12391.33 ± 3075.59	13190.42 ± 3342.41	0.414	0.102
Masseter muscle cross-sectional area (mm^2^)	419.58 ± 79.24	460.38 ± 70.87	0.079	391.54 ± 59.41	405.70 ± 62.78	0.447	0.191
Masseter muscle length (mm)	38.96 ± 3.83	46.18 ± 3.49	< 0.001	44.39 ± 3.21	42.98 ± 3.26	0.149	<0.001
Masseter muscle width (mm)	13.51±2.09	10.81±1.31	< 0.001	12.04±1.39	12.69±2.37	0.268	0.009

^*^p<0.05 paired *t* test

### Factors Related to Changes in the Masseter Muscle Volume and Cross-Sectional Area

In addition, DFFM, occlusal plane angle and U1-L1 deviation significantly decreased from T1 to T2 (*P *< 0.05) (Table 3). Linear regression was performed on the factors that were related to the volume and cross-sectional area of both sides of the masseter muscle. The occlusal plane angle was significantly related to changes in the cross-sectional area of the masseter muscle. A smaller occlusal plane angle was significantly associated with a smaller cross-sectional area of the masseter muscle (Table 4).

**Table 3:** Morphological factors in the facial asymmetry at T1 and T2

Morphological factors	T1	T2	*P*-value
DFFM at Menton (mm)	8.95±3.03	2.13±1.20	< 0.001
Ul-Ll deviation (mm)	7.42±2.84	3.14±0.67	< 0.001
Occlusal plane angle (°)	9.04±4.70	3.13±3.01	< 0.001

^*^p < 0.05 paired t test

**Table 4:** Factors related to change of volume and cross-sectional area based on linear regression analysis

Morphological factors	Dev		N-Dev	
	r2	*P-*value	r2	*P-*value
DFFM at Menton (mm)	0.034	0.413	0.047	0.334
Ul-Ll deviation (mm)	0.034	0.410	0.021	0.521
Occlusal plane angle (°)	0.189	0.043	0.207	0.033

## Discussion

It is important to establish skeletal stability for orthognathic surgery. Changes in skeletal stability and occlusion can result in changes in the masticatory force vectors and occlusal force. The relationship between masticatory muscles and maxillofacial morphology needs to be studied. The purpose of the current study was to investigate the morphological changes in the masseter muscles of patients with facial asymmetry after orthognathic surgery and to provide objective data for postoperative effect evaluation. In addition, changes in the morphology of the masseter muscle were also analyzed. Pre- and postoperative CT data were used to generate a masseter muscle model. Therefore, further research to investigate the underlying mechanism of changes in facial morphology related to surgery is needed.

Precise quantitative measurements are critical for evaluating the morphological characteristics of the masseter muscle. We first evaluated the volume of masseter muscles to determine whether there was a difference between the deviated side and non-deviated side in patients with facial asymmetry. The volume of the masseter muscles on the non-deviated side was greater than that on the deviated side before and after surgery. However, at 1-year postoperatively, the volume of the masseter muscle had decreased bilaterally. The cross-sectional area of the masseter muscle was analyzed as well, because it is reported to be closely correlated with volume [18]. In this study, the result revealed that the cross-sectional area of the masseter muscle on both sides had the same tendency with the masseter muscle volume did, indicating that both items were sensitive to the alterations that occur during the orthognathic surgery. The changes occurred in the volume and cross-sectional area of masseter muscle are the results of impaired oral function and decreased postoperative masticatory function. The maximal occlusal force decreases after orthognathic surgery, and subsequently improves, but is still lower than that in normal subjects [17, 19, 20]. The size of a muscle is directly related to its capacity to generate maximum tension, and the length of its lever arm relative to the mandibular condyle determines the maximum torque that the muscle can exert [12]. Therefore, the cross-sectional area of masseter muscles is correlated with the maximum occlusal force and a decreased masseter muscle cross-sectional area could lead to decreasing masticatory function. We also found that during surgery, the volume and cross-sectional area of the masseter muscle on the non-deviated side were greater. This may be because the skeleton of the non-deviated side moved further posteriorly due to surgery, resulting in a more marked decrease in the volume and cross-sectional area. Some studies have shown that subjects with long faces arrested development of the jaw muscle, which manifests as not only a reduction in the cross-sectional area but also a decrease in intrinsic muscle strength [12]. If this suggestion is supported, it could be hypothesized that a greater reduction in cross-sectional area or intrinsic muscle strength may lead to asymmetry on the longer side of the mandible in patients with facial asymmetry. Additionally, this study revealed that the discrepancies in volume and cross-sectional area between the deviated side and the non-deviated side decreased after the operation, although no statistically significant findings were observed. The disparities in the length and width of the bilateral masseter muscle significantly decreased before and surgery. From the above results, we can infer that the volume and morphology of masseter muscle are asymmetric in patients with facial asymmetry, which may be one of factors causing facial asymmetry. However, masseter muscle asymmetry could be improved with maxilla-mandible correction, suggesting that variations in the soft tissue of the masseter muscle are associated with the properties of facial morphology.

When we analyzed these morphological factors, a significant correlation between the cross-sectional area and occlusal plane angle was observed in this study. The occlusal plane angle was positively related to the cross-sectional area of the masseter muscle, which indicated an association between the degree of occlusal plane inclination and changes in the masseter muscle. Greater atrophy of the masseter muscle was observed during orthognathic surgery in patients with decreased occlusal plane angle, which is due to improved dental compensation in patients with facial asymmetry. The results of the present study suggest that particular attention should be given to the masseter muscle in patients with large occlusal plane angles, because orthognathic surgery has a more obvious effect on atrophy of the masseter muscle. However, the degree of mandibular deviation was not significantly associated with the cross-sectional area of the deviated side and non-deviated side of the masseter muscle, which differed from findings of a previous study [21]. The differences in the study findings may result from differences in measurement methods used to evaluate facial asymmetry. Consequently, the Menton position did not have much influence on the loss of soft tissue during the surgery.

We acknowledge that there were several limitations in our study. The work presented herein is still limited due to its nature as a relatively short-term follow-up study. There was only one postoperative follow-up time point, and one year of follow-up was not sufficient to show postoperative stability. Future investigations should focus on evaluating long-term postoperative stability. The sample size was relatively small and a prospective study with a larger sample size changes in the muscles responsible for mastication assessing changes in the muscles responsible for mastication in patients with facial asymmetry is needed in the future. Moreover, further investigations analyzing other masticatory muscles such as medial pterygoid muscles, which are responsible for biting force, are needed.

## Conclusion

Our study revealed that changes in the masseter muscle and jaw asymmetry are interrelated. Bilateral differences in morphology of the masseter muscles were significantly reduced after orthognathic surgery in patients with facial asymmetry. These findings suggest that masseter muscle asymmetry can be improved according to the new skeletal structure and position. Additionally, it appears that the occlusal plane angle has a greater effect on masseter muscle asymmetry than Menton position does. Thus, we should pay attention to postoperative masseter muscle changes even after occlusal plane angle adjustment and not only facial midline correction when planning orthognathic surgeries.
