# Correction: Changes in 3-Dimensional Measurements of Masseter Muscle After Orthognathic Surgery in Patients with Facial Asymmetry

**DOI:** 10.1007/s00266-024-04357-8

**Published:** 2024-09-03

**Authors:** Xiaobin Yang, Zhengguo Piao, Yaoran Liu, Lunqiu Chen, Luo Huang

**Affiliations:** 1 Department of Oral and Maxillofacial Surgery, Guangzhou Key Laboratory of Basic and Applied Research of Oral Regenerative Medicine, Affiliated Stomatology Hospital of Guangzhou Medical University, Guangzhou, 510150 China; 2 Key Laboratory of Oral Medicine, Guangzhou Institute of Oral Disease, NO.39 Huangsha Avenue, Guangzhou, 510150 China

**Correction to: Aesth Plast Surg** 10.1007/s00266-024-04309-2

The original online version of this article was revised to correct the presentation of the name of coauthor Zhengguo Piao.

The original article has been corrected.

